# A Silicon Optical Bench-Based Forward-View Two-Axis Scanner for Microendoscopy Applications

**DOI:** 10.3390/mi11121051

**Published:** 2020-11-28

**Authors:** Dong Zheng, Dingkang Wang, YK Yoon, Huikai Xie

**Affiliations:** 1 Department of Electrical & Computer Engineering, University of Florida, Gainesville, FL 32611, USA; noplaxochia@ufl.edu (D.W.); ykyoon@ece.ufl.edu (Y.Y.); 2 School of Information and Electronics, Beijing Institute of Technology, Beijing 100000, China; hk.xie@ieee.org

**Keywords:** optical coherence tomography, MEMS mirror, forward-view scanning, squamous dysplasia (SD), lung cell carcinoma

## Abstract

Optical microendoscopy enabled by a microelectromechanical system (MEMS) scanning mirror offers great potential for in vivo diagnosis of early cancer inside the human body. However, an additional beam folding mirror is needed for a MEMS mirror to perform forward-view scanning, which drastically increases the diameter of the resultant MEMS endoscopic probe. This paper presents a new monolithic two-axis forward-view optical scanner that is composed of an electrothermally driven MEMS mirror and a beam folding mirror both vertically standing and integrated on a silicon substrate. The mirror plates of the two mirrors are parallel to each other with a small distance of 0.6 mm. The laser beam can be incident first on the MEMS mirror and then on the beam folding mirror, both at 45°. The MEMS scanner has been successfully fabricated. The measured optical scan angles of the MEMS mirror were 10.3° for the x axis and 10.2° for the y axis operated under only 3 V. The measured tip-tilt resonant frequencies of the MEMS mirror were 1590 Hz and 1850 Hz, respectively. With this compact MEMS design, a forward-view scanning endoscopic probe with an outer diameter as small as 2.5 mm can be made, which will enable such imaging probes to enter the subsegmental bronchi of an adult patient.

## 1. Introduction

**Figure 1:**
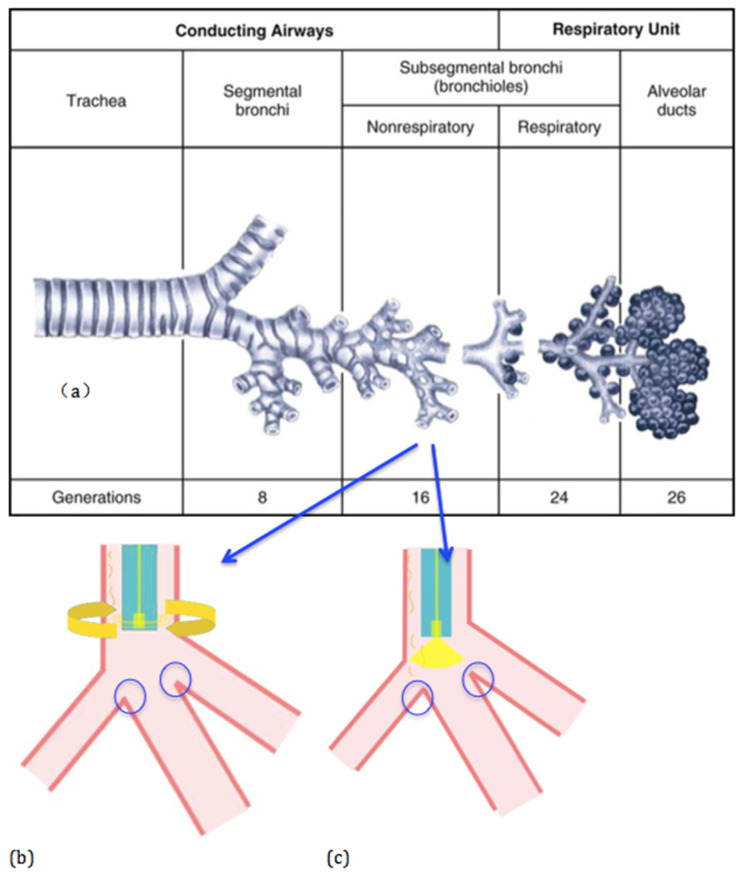
(**a**) The lung structure showing the multi-level bronchi 1. (**b**) Imaging with a side-view probe. (**c**) Imaging with a front-view probe.

Squamous dysplasia of the lung (SD) has been widely considered as a pre-invasive lesion leading to lung squamous cell carcinoma (SCC) [1]. Figure 1a shows the anatomy structure of the lung, which consists of multiple levels of bronchi [1]. Different from other types of lung cancers, SCC is known to originate from low-level bronchi such as subsegmental bronchi [2]. As such, SD in this article refers to the SD in subsegmental bronchi so SD in subsegmental bronchial branches is the focus of this work. The inner diameters of subsegmental bronchi of adults are about 2–3 mm [3,4,5]. To this date, SD is still not detectable using common medical imaging modalities such as X-ray, ultrasound imaging, CT and MRI due to their inadequate resolutions [6,7]. Thus, SD detection must rely on a biopsy, which is an invasive and time-consuming procedure and poses a high risk to patients. Therefore, non-invasive biomedical imaging techniques with a high resolution are needed to detect SD by safe, fast and accurate means [2]. Fortunately, optical coherence tomography (OCT) has emerged as such an imaging technique [8,9,10,11,12] OCT typically employs near infrared light that is radiation-free and safe. The question becomes how to bring the OCT’s capability inside the human body for in vivo diagnosis, i.e., how to make miniature OCT endoscopic imaging probes that can be inserted down to subsegmental bronchi.

Microelectromechanical system (MEMS) technology has been changing our daily life by enabling numerous smart functions (e.g., smart phones and autonomous driving) with sensors and actuators that are small and inexpensive [8]. MEMS has been used to miniaturize OCT probes for two decades [13,14,15,16,17,18]. The first MEMS OCT probe was developed in 2001 by incorporating an electrothermal one-axis scanning MEMS mirror, demonstrating the feasibility of combing MEMS and OCT techniques for endoscopic imaging [13]. Since then, researchers have developed various electrothermal MEMS mirrors and applied them to endoscopic OCT, leading to OCT probes with smaller probe diameters [19,20] but almost all of those probes are side-viewing. When the probe diameter is below 3 mm, it suggests that this kind of probe can then reach the subsegmental bronchi to detect SD. However, as shown in Figure 1b, only the side wall of a bronchus can be imaged if a side-viewing probe is employed, meaning it is unable to see the juncture leading to the next level bronchi. It will therefore be extremely challenging to guide the probe into the bronchus of interest and will also pose a high risk of damaging the branch juncture. On the other hand, a front-view probe can detect the juncture, as shown in Figure 1c. Thus, forward-view OCT probes with an outer diameter of less than 3 mm are needed.

Fu et al. designed a small front-view OCT probe with an outer diameter of 0.7 mm but this probe had no scanning ability [6]. Li et al. developed a scanning OCT probe with an outer diameter of 1 mm but this probe provided only one-axis scanning and a low resolution of 60 μm [21]. Duan et al. reported a front-view MEMS OCT probe with two-axis scanning and a resolution of about 10 μm but the probe’s outer diameter was as large as 5 mm due to the need to assemble a prism to direct the light scanning forward [22].

In this work, an integrated forward-view MEMS scanner design is proposed to solve the problems described above. In this novel design, two mirrors are integrated on a single silicon substrate, i.e., a silicon optical bench (SiOB). The first mirror folds the optical beam and the second mirror scans the optical beam in two axes. This MEMS dual-mirror design completely eliminates the need for assembling a prism or a beam folding mirror, enabling OCT probes with much reduced outer diameters. This paper is organized as follows. Section 2 introduces the MEMS dual-mirror concept and enabled probe design while Section 3 presents the design of the MEMS dual-mirror. Section 4 describes the MEMS fabrication process. Section 5 presents the characterization results of the fabricated MEMS scanner and the first attempt of assembling a forward-scanning probe. Finally, a summary is given at the end.

## 2. Forward-View Scanning Probe Concept

**Figure 2:**
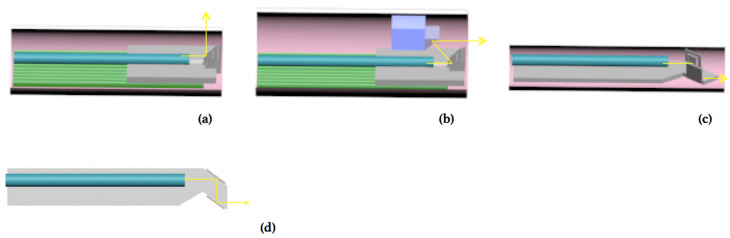
Various microelectromechanical system (MEMS) probe designs. (**a**) A typical side-view scanning probe. (**b**) A typical forward-view probe with a beam folding mirror to fold the optical beam. (**c**) A novel forward-view probe with two vertical mirrors on a silicon substrate. (**d**) A top view of the novel forward-view probe, showing the light path inside the probe.

**Figure 3:**
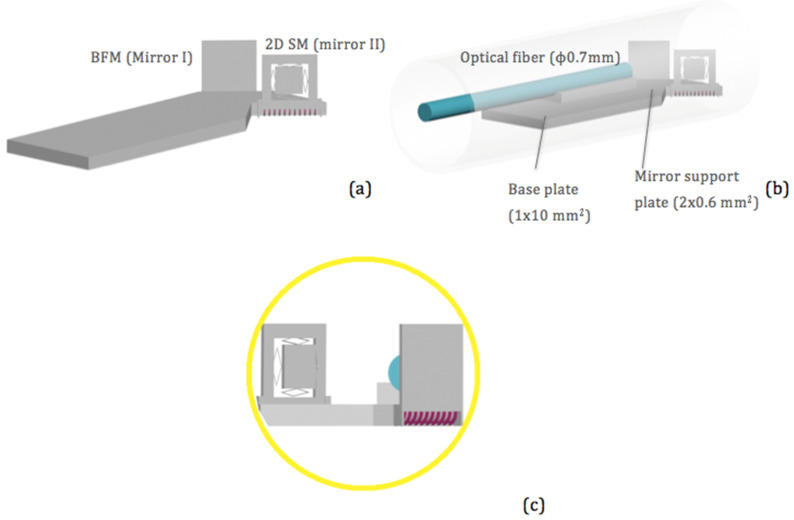
(**a**) 3D model of the proposed probe. (**b**) The dimensions of the silicon optical bench (SiOB) based vertical MEMS dual-mirror. (**c**) End view of the probe.

The structure of a side-view MEMS scanning probe is illustrated in Figure 2a, where an optical beam coming from an optical fiber reached the MEMS mirror plate that bounced off the optical beam towards the side of the tube. If a reflective beam folding mirror was inserted on the optical path, folding the optical beam forward, then a forward-view MEMS scanning probe could be obtained, as shown in Figure 2b. The outer diameter (OD) of the forward-view probe was much larger than that of the side-view probe due to the extra space occupied by the added beam folding mirror. If we integrated both the beam folding mirror and the MEMS mirror vertically standing on a single substrate, as shown in in Figure 3a, the probe diameter could be greatly reduced, as illustrated in Figure 2c, and, at the same time, the assembly simplified.

It was very challenging to make a forward-view MEMS endoscopic probe with an outer diameter (OD) smaller than 5.0 mm based on the design shown in Figure 2b [22]. In contrast, by using the vertical MEMS dual-mirror design shown in Figure 3a, the probe OD could be reduced by about one half. A 3D model of the proposed new probe design is shown in Figure 3b. The probe consisted of a single-mode fiber, a graded-index (GRIN) lens and a vertical MEMS dual-mirror chip. The optical fiber was aligned to the GRIN lens through a V-groove block. The vertical MEMS dual-mirror chip was a monolithic integration of a vertical beam folding mirror, a vertical two-axis scanning mirror and a silicon optical bench (SiOB). As shown in Figure 3b, the fiber-GRIN lens module was glued directly on the SiOB where the vertical two-axis scanning mirror was 45° to the optical axis and the vertical beam folding mirror was parallel to the two-axis scanning mirror. Thus, the optical beam was first coupled into the optical fiber, collimated by the GRIN lens incident on, scanned by the two-axis scanning mirror and then folded forward by the beam folding mirror. The outer frames of the two-axis scanning mirror (marked as Mirror II) and the beam folding mirror (marked as Mirror I) were both 1.2 mm × 1.2 mm. The distance between these two mirrors was 0.6 mm. Through a simple geometric calculation, we found that the height and width of this scanning module were 1.4 mm and 2 mm, respectively. Thus, this scanning module could fit in a probe with an inner diameter of 2.28 mm. If the wall thickness of the probe was 0.1 mm, then the outer diameter of the probe would be 2.5 mm, as shown in the end view of the probe in Figure 3c. The SiOB based vertical MEMS dual-mirror was the enabling component and thus is the focus of this paper that will be discussed in detail in the next two sections.

## 3. SiOB Based Vertical MEMS Dual-Mirror Design

**Figure 4:**
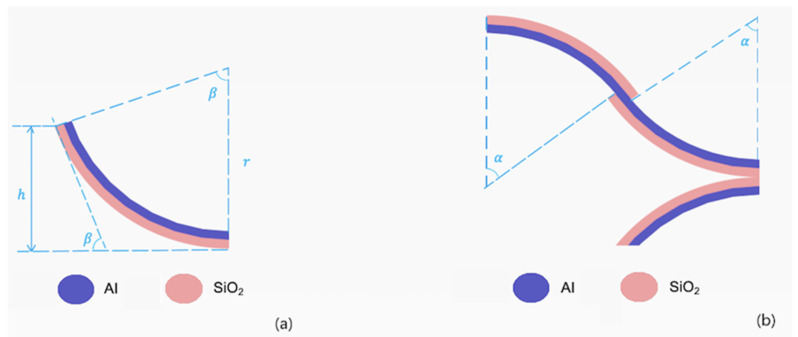
Illustration of the electrothermal bimorphs. (**a**) An Al/SiO_2_ bimorph cantilever. (**b**) A double S-shaped inverted-series-connected (ISC) bimorph actuator.

As shown in Figure 3a, the first mirror was a fixed non-scanning flat mirror and the second one was a 2D scanning mirror. The 2D scanning mirror was supported by four pairs of electrothermal bimorph actuators with one pair on each side of the mirror plate. The basic structure of the electrothermal bimorph actuators was a bimorph cantilever, as shown in Figure 4a, which consisted of two layers made of two materials with different coefficients of thermal expansion (CTEs). The radius of the curvature of the bimorph could be changed by changing the bimorph temperature through a resistive heater embedded in the bimorph. In order to cancel the tip-tilt and lateral shift of a simple bimorph actuator, a double S-shaped, inverted-series-connected (ISC) bimorph actuator design was developed by Todd and Xie [23]. The same ISC bimorph actuator design, as illustrated in Figure 4b, was adopted in this 2D scanning mirror. The two bimorph materials employed were Al and SiO_2_ and the resistive heater was made of Pt.

**Figure 5:**
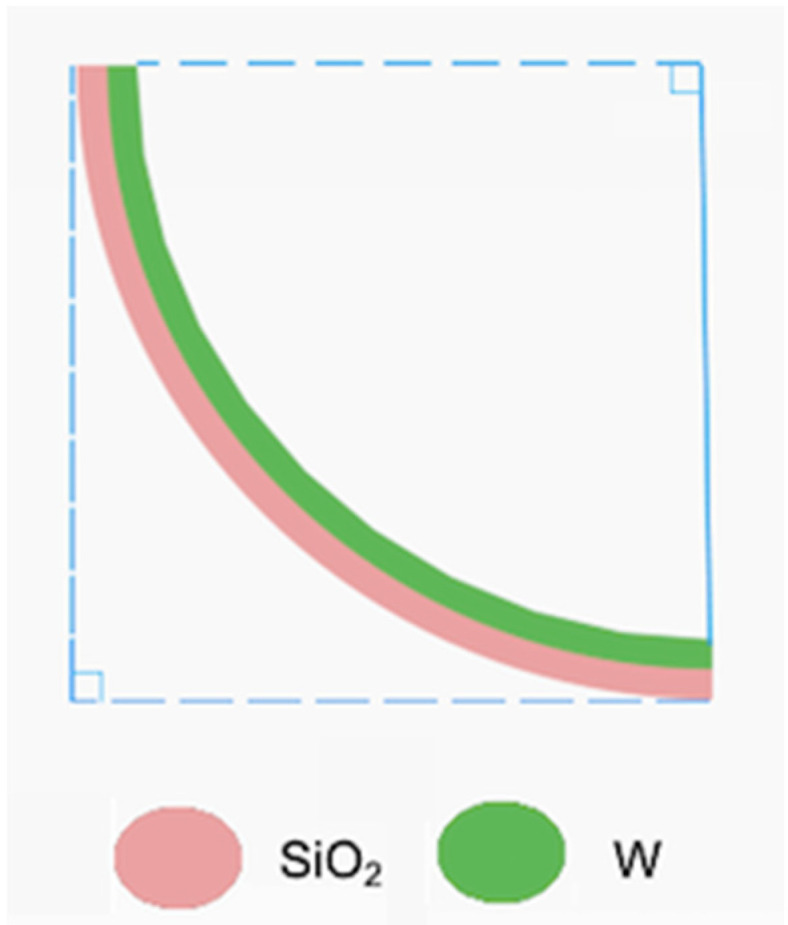
A W/SiO_2_ vertically-bent bimorph beam used in a 90° tilting structure.

As shown in Figure 3a, both mirrors were tilted 90° out of the silicon substrate, i.e., a silicon optical bench (SiOB). The SiOB provided the mechanical support and the electrical wiring and pads. The vertical orientation of the mirror plates was achieved via vertically-bent bimorph beam arrays. As shown in Figure 5, the vertically-bent bimorph beams consisted of tungsten (W) and SiO_2_ where the W layer was on top of the SiO_2_ layer. After being released, the bimorph beams bent towards the W side, forming a 90° tip angle when a proper beam length was chosen. The reason to use W instead of Al for these bimorph beams was two-fold. Firstly, W films can be sputtered with high stresses up to a few gigapascals (a unit of pressure measurements), which are at least one order of magnitude greater than those of Al films. Thus, the bimorph beams can be short and stiff. Secondly, W has a much higher Young’s modulus than Al, again making the bimorph beams even stiffer.

**Figure 6:**
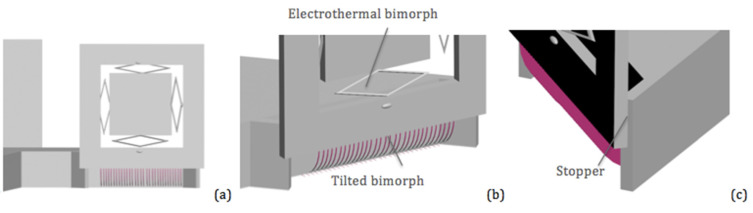
Schematic view of the mirror’s tilting structure. (**a**) 2D MEMS mirror design. (**b**) A side view of the mirror’s tilting structure. (**c**) A closer view of the mirror’s tilting structure with the stoppers.

The 2D MEMS mirror design is shown in Figure 6a where the 2D scanning mirror (2D SM) plate was 0.7 mm by 0.7 mm and the ISC actuation bimorph beams were 18 μm wide. The actual designs of the vertical bending structures are shown in Figure 6b. Each mirror frame was supported by an array of bending bimorphs and the bimorphs were composed of a 0.51 μm-thick W layer and a 1 μm-thick SiO_2_ layer. The ratio between the two thicknesses was 1.96, which is the square root of the inverse ratio between the Young’s moduli of W and SiO_2_. Each bending bimorph was 22 μm wide. With an estimated radius of curvature of about 250 μm, the length of the bending bimorphs needed to be about 360 μm to form the 90° bending angle. In this design, the length was set to a greater value (e.g., 400 μm) that allowed the bending bimorphs to reach a bending angle of over −90° (e.g., 100°). A stopper structure was then implemented to confine the bending angle to 90°. Figure 6c shows the design of the stopper structure.

As shown in Figure 3c and Figure 6a, the two most critical factors that influenced the height of the scanning module were the height of the mirror frame and the radius of the curvature of the bending bimorphs. The radius of the curvature of the bending bimorphs was determined above, which was 0.25 mm. Thus, according to Figure 3c, the height of the 2D MEMS mirror frame must not exceed 1.2 mm in order to keep the OD of the probe no larger than 2.5 mm. The outer frame of Mirror II was therefore chosen as 1.2 mm × 1.2 mm.

**Figure 7:**
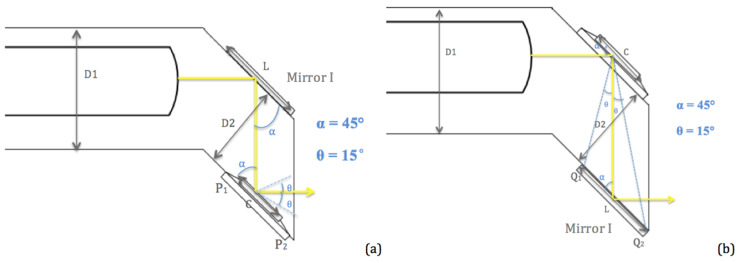
Top view of the MEMS chip structure. (**a**) A structural design with the beam folding mirror as the first incident mirror. (**b**) A structural design with scanning mirror as the first incident mirror.

Figure 7 shows the top view of the entire MEMS chip design. The width of the mirror support plate, D2, was carefully chosen to ensure the optical beam to be relayed from Mirror I to Mirror II was without beam truncation. D2 must satisfy:

(1) $$D2+\frac{L}{2}<L_{2}<W+\frac{L}{2}+C$$

where *L* is the length of Mirror I, *c* is the length of Mirror II and $L_{2\;}$ is the pre-determined length of the mirror support plate through the height of the mirror and the radius of the probe. Therefore, the length of the mirror support plate ($L_{2\;}$) should not exceed 2 mm to fit inside the probe. The two sides of the inequation represented the extremum of the width of Mirror II, P_1_ and P_2_. The left side of the inequation ensured that the light at least reached P_1_ and the right side of the inequation indicated that the light did not exceed P_2_. In this way, the limited length of D2 guaranteed that the light beam reached Mirror II to produce an accurate imaging scan.

In order to ensure the light coming out of the tube, D2 also must satisfy the requirement of not letting the tube block the light path. This can then be represented as:

(2) $$\frac{D2}{\sin45{}^{\circ}}\cdot \sin\theta +\frac{L}{2}+W<L_{2}.$$

Only under these two conditions does the light come out of the tube without truncation and/or blocking after reflecting once on each of the mirrors.

Figure 7 represents the two different structures of the chip. Figure 7a shows the chip structure as designed, with the beam folding mirror as Mirror I. Figure 7b shows the chip structure with a switch of the mirrors in which a 2D scanning mirror was the new Mirror I. With the conditions calculated above under the design of Figure 7a, the switch of mirrors did not affect the path of the light beam. This meant that the light reached Q_1_ and did not exceed Q_2_ to be reflected out of the probe. The reason to have two different structure designs was to determine and reduce the difficulty in the assembling process. Each design has its own pros and cons based on the perception of the assembling person. The result of the assembling process shows the design demonstrated in Figure 7b was easier to assemble, thus this design was being used to show later results.

The base plate for the optical fiber and the GRIN lens was a 0.5 mm-thick SOI wafer. The width of the base plate was 1.95 mm, determined by the mirror size and the light path. The length of the base plate was 10 mm, which was chosen according to the convenience of assembling the optical fiber and the GRIN lens in the later process.

## 4. Device Fabrication

**Figure 8:**
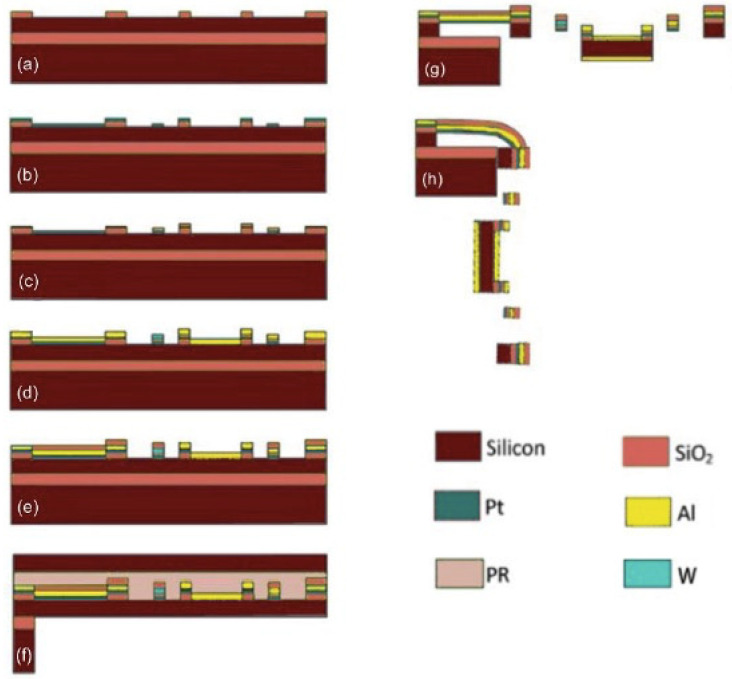
MEMS fabrication processing. (**a**) Mirror frame deposit. (**b**) Pt heating sputtering. (**c**) W layer sputtering. (**d**) Connection layer sputtering. (**e**) Protecting and pad layer deposit. (**f**) Backside etching. (**g**) MEMS structure etching. (**h**) Final release.

The device was fabricated on an SOI wafer with a 30 µm-thick device layer, a 2 µm-thick buried oxide (BOX) layer and a 500 µm-thick handling layer. The fabrication process is sketched in Figure 8. In the first step (Figure 8a), a 1 µm-thick SiO_2_ layer was deposited on the device layer via plasma enhanced chemical vapor deposition (PECVD) followed by photolithography and wet etching to form a slope for the next metal layer to achieve a smooth step coverage. In the next step, as shown in Figure 8b, a 0.2 μm-thick Pt layer was sputtered. Note that a 100 nm-thick SiO_2_ layer was deposited before the Pt sputter to provide the electrical insulation to the silicon substrate. A 0.51 μm-thick tungsten (W) layer was then sputtered and patterned (Figure 8c). During the W sputtering, Ar pressure was carefully tuned and maintained at 6.5 mTorr to produce high stress W films. After that, a 1.1 μm-thick Al layer and a 1 μm-thick SiO_2_ layer were sputtered and patterned consecutively (Figure 8d,e). At this point, all of the steps on the front side of the SOI wafer were completed. The front side was then spin coated with a 2 µm-thick AZ1512 and baked to protect the patterned structures on the front side during the back side processing.

The back side processing started with a 14 µm-thick photoresistant coating using multiple AZ1512 layers. After photolithography, a deep reactive ion etch (DRIE) was employed to etch through the handle layer with straight silicon sidewalls and then a reactive ion etch (RIE) to remove the BOX layer (Figure 8f). At this point, the devices were ready for release. The release process was done from the front side in which an anisotropic DRIE was first done to etch the silicon between those bimorph beams and then an isotropic DRIE was performed to undercut the silicon under the bimorph beams (Figure 8g). When the silicon undercut was complete, the bimorph beams bent so that the MEMS mirror popped up and erected vertically on the silicon substrate (Figure 8h).

**Figure 9:**
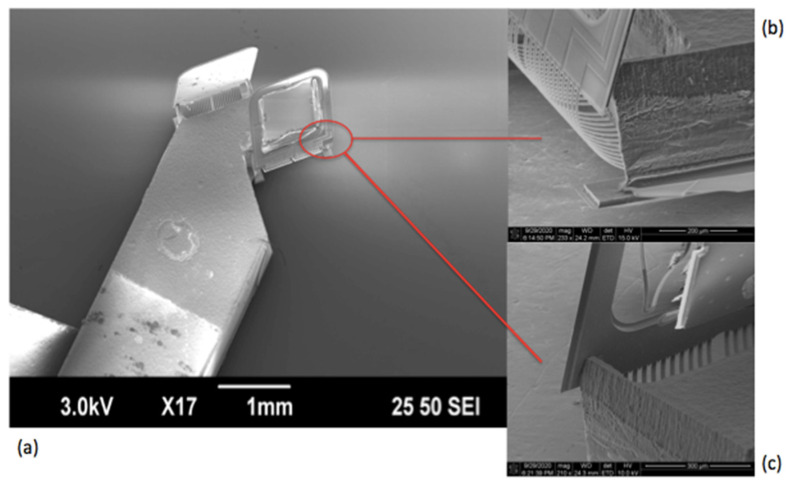
SEMs of a fabricated device. (**a**) Top view of the assembled MEMS chip structure. (**b**,**c**) Detailed stopper structure.

Figure 9a shows a scanning electron micrograph (SEM) of a fabricated device, where both Mirror I and Mirror II are stood up on the silicon substrate. However, both mirrors were slightly off from the perfect 90° vertical angle. This tilt was mainly caused by the process imperfections including the silicon sidewall slope and the small silicon undercut under the stopper structures. The size of the 2D MEMS mirror plate was 0.7 × 0.7 × 0.03 mm^3^ and the mirror support plate was 2 × 0.6 × 0.53 mm^3^. Close-up views of an ISC bimorph actuator, the vertically bending bimorphs and the stopper are shown in Figure 9b,c, respectively. The resistances of the actuators were between 450–470 Ω.

## 5. Device Assembly and Characterization

**Figure 10:**
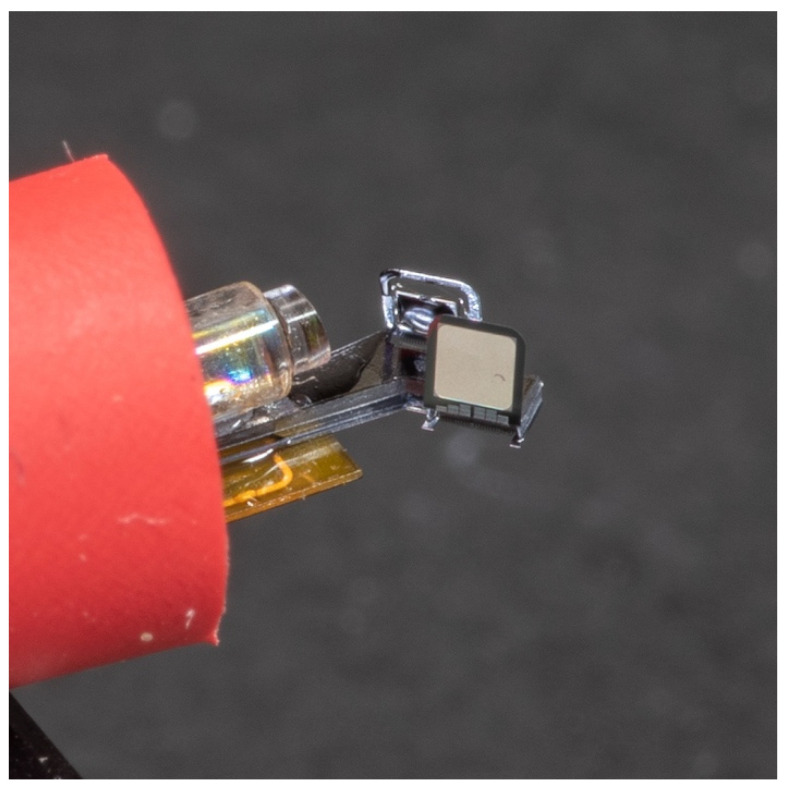
Photo of an assembled probe.

In order to test its functions, the MEMS device must be packaged first. A fiber-GRIN lens module was aligned and fixed on the silicon substrate of the MEMS device using a UV glue. There were electrical pads on the back side of the MEMS device. Thus, the MEMS device was directly placed on a flexible printed circuit board (FPCB) designed with the corresponding pads for electrical connection. Silver epoxy was used as the conductive glue. An assembled device is shown in Figure 10. The red outer tube of the device was added for illustrative purposes. The probe size could be smaller in any actual implementation. The location of the fiber-GRIN lens assembly shown in Figure 10 was slightly adjusted to compensate for the fabrication error that occurred during the MEMS fabrication.

**Figure 11:**
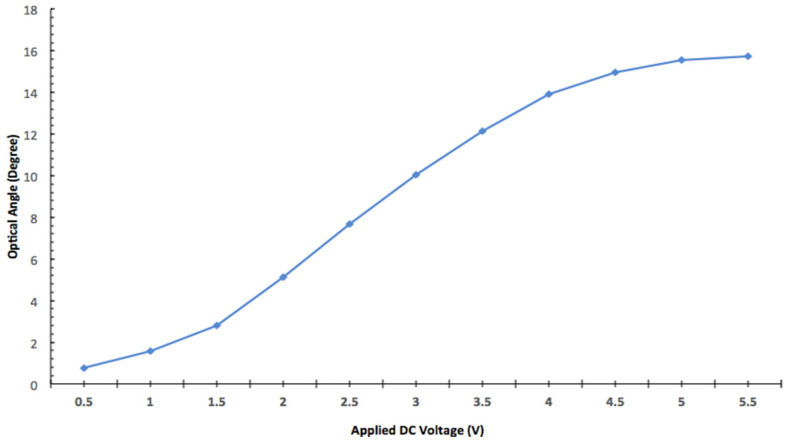
Quasi-static angular response of the MEMS mirror. Measurement errors: 0.1°.

For testing purposes, a 635 nm HeNe laser was coupled into the fiber. The laser beam was relayed by the two vertical mirrors on the SiOB. The scan angle was calculated by measuring the moving distance of the laser spot on a screen. The quasi-static scan response of one ISC bimorph actuator is shown in Figure 11. Its maximum optical scan angle reached ± 15.9° at 5 V. The response showed a good linear relationship from 1.5 V to 4 V.

**Figure 12:**
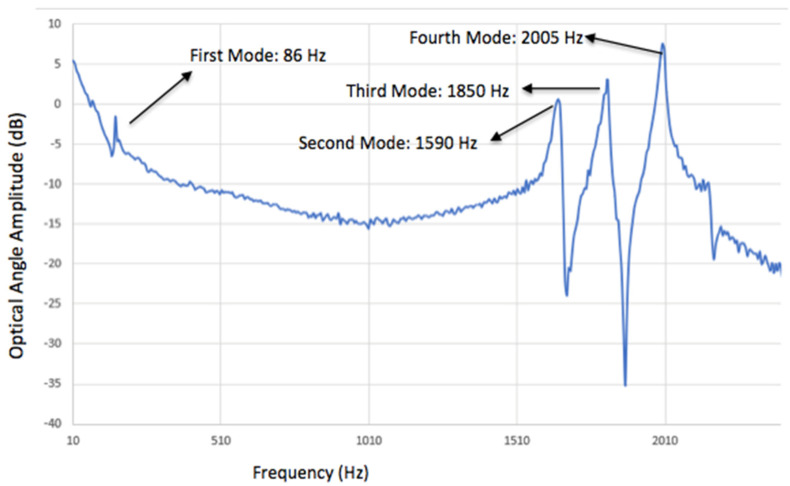
Frequency response of the MEMS device.

The frequency response was also measured with a position-sensitive detector (PSD), which is shown in Figure 12. The first mode occurred at 86 Hz, which was the frame rotation mode. The second mode was a piston mode occurring at a resonant frequency of 1590 Hz. The third mode occurred at 1850 Hz, which was the tip-tilt angular scan mode. The fourth mode was the longitudinal rotational mode, occurring at 2005 Hz. Due to the fabrication variations, the measured resonant frequencies had a smaller deviation than the simulation data.

## 6. Conclusions

In this work, a prototype of an electrothermal MEMS probe was developed. This MEMS probe could produce large two-dimensional scans at low driving voltages. Its diameter could be used in subsegmental bronchial tubes and provided sufficient scanning range. The probe could meet the precise scanning of the epithelial cells of the tracheal wall and also provide influence parameters for its localization. This kind of electrothermal MEMS probe has a great application potential in bronchoscopic image diagnosis. As mentioned above, the new MEMS design could fulfill expected requirements.

The next step is to improve the mirror structure by switching the position of Mirror II and Mirror I and reducing the size of Mirror I to further decrease the outer diameter of the probe to 2.5 mm. This device could help to expand a new type of ultra-compact forward-scanning microendoscope optical imaging probes for in situ early cancer detection.
